# Bidirectional association between nonalcoholic fatty liver disease and type 2 diabetes in Chinese population: Evidence from the Dongfeng-Tongji cohort study

**DOI:** 10.1371/journal.pone.0174291

**Published:** 2017-03-28

**Authors:** Yaru Li, Jing Wang, Yuhan Tang, Xu Han, Bing Liu, Hua Hu, Xiulou Li, Kun Yang, Jing Yuan, Xiaoping Miao, Ping Yao, Sheng Wei, Youjie Wang, Yuan Liang, Xiaomin Zhang, Huan Guo, An Pan, Handong Yang, Frank B. Hu, Tangchun Wu, Meian He

**Affiliations:** 1 Department of Occupational and Environmental Health and State Key Laboratory of Environmental Health for Incubating, School of Public Health, Tongji Medical College, Huazhong University of Science and Technology, Wuhan, Hubei, China; 2 Dongfeng Central Hospital, Dongfeng Motor Corporation and Hubei University of Medicine, Shiyan, Hubei, China; 3 Departments of Nutrition and Epidemiology, Harvard School of Public Health, Boston, Massachusetts, United States of America; Universita degli Studi di Verona, ITALY

## Abstract

**Objectives:**

The aim of this study is to examine the bidirectional association between nonalcoholic fatty liver disease (NAFLD) and type 2 diabetes mellitus (T2DM).

**Methods:**

The data was derived from the Dongfeng-Tongji cohort study, which was established in 2008 and followed until October 2013. NAFLD was classified as none, mild, moderate/severe based on ultrasound examination. The analysis to examine the association between NAFLD and incident T2DM risk included 18,111 participants free of diabetes at baseline and the duration of follow-up was 4.60 ± 0.60 years. Cox proportional regression model was used to calculate the hazard ratio (HR) for the association. The analysis to investigate the association between T2DM and incident NAFLD risk included 12,435 participants free of NAFLD at baseline. Logistic regression model was used to calculate the odd ratio (OR) of NAFLD.

**Results:**

Compared with those without NAFLD, individuals with mild or moderate/severe NAFLD had a monotonic elevated risk of developing T2DM (HR: 1.88 [95% CI: 1.63–2.18] and 2.34 [1.85–2.96], respectively) after adjustment for potential confounders. In a parallel analysis, compared to participants with fasting plasma glucose < 6.1 mmol/L, the ORs of developing NAFLD in subjects with impaired fasting glucose and T2DM were 1.35 (95% CI: 1.16–1.57) and 1.40 (95% CI: 1.22–1.62), respectively.

**Conclusions:**

Our results provide compelling evidence that the NAFLD-T2DM association is bidirectional in Chinese population.

## Introduction

The prevalence of type 2 diabetes mellitus (T2DM) increases worldwide. A recent national survey reported that the prevalence of diabetes and prediabetes among a representative sample of Chinese adults was 11.6% and 50.1%, respectively[1]. The prevalence of diabetes is much higher in those aged ≥ 70 years (23.5%) and those with body mass index (BMI) ≥ 30.0 kg/m^2^ (24.5%) [1].

Meanwhile, nonalcoholic fatty liver disease (NAFLD) is an alarming public health problem and one of the main causes of chronic liver disease. The diagnosis of NAFLD requires evidence of fatty liver in nonalcoholic individuals and free from alternative etiology of steatosis [2]. NAFLD is histologically further categorized into nonalcoholic fatty liver (NAFL) and nonalcoholic steatohepatitis (NASH) [2]. It is indicated that obesity, diabetes, dyslipidemia, and hypertension were associated with the development of NAFLD [3]. The prevalence of NAFLD varies dramatically among different populations due to the differences in diagnostic tools and study populations. The prevalence of NAFLD is 20–30% in worldwide [4] and 20.1% in Chinese adults [5]. T2DM and NAFLD have numerous common risk factors and several studies examined the association between NAFLD and T2DM [6]. The comorbidity of T2DM in NAFLD patients has been observed in several studies. A systematic review and meta-analysis showed that NAFLD, as diagnosed by either liver enzymes or ultrasonography, significantly increased the risk of incident T2DM and metabolic syndrome (MetS) [7]. Studies in Japanese adults reported that non-overweight individuals with NAFLD had increased risk of incident T2DM [8]. In addition, several longitudinal studies explored the association between NAFLD improvement/remission and T2DM incidence reduction [9,10]. Meanwhile, a cross-sectional study indicated that the prevalence of NAFLD was significantly higher in T2DM patients [11]. A retrospective cohort study showed that adults with diabetes had increased risk of incident diabetic hepatopathy [12].

Although growing evidence suggested that the NAFLD-T2DM relation is bidirectional [13–15], few studies simultaneously investigated the bidirectional association between NAFLD and T2DM in a prospective setting [16]. Therefore, in the present study, we aimed to address the bidirectional relationship between NAFLD and T2DM in a prospective cohort study among a middle-aged and elderly Chinese population.

## Methods

### Study population

The Dongfeng-Tongji cohort (DFTJ cohort) study was established in 2008 and conducted by Tongji Medical College, Huazhong University of Science and Technology and Dongfeng Motor Corporation (DMC). All participants were retired employees of DMC. Physical examinations were performed by trained physicians, nurses, and technicians in Dongfeng Central Hospital. The DFTJ cohort study investigated a wide range of lifestyle, dietary, psychosocial and occupational factors and biochemical factors in relation to the development of chronic diseases. We recruited 87% (n = 27,009 out of 31,000) of retired employees who agreed to answer the questionnaire information and provided baseline blood samples between September 2008 and June 2010. The participants will be followed up every five years. The first follow-up was conducted from April to October in 2013 and recruited 38,295 participants. Among them, 25,978 participants were from the 2008 baseline data (27,009 participants) and the follow-up rate is 96.2%. The study has been approved by the Medical Ethics Committee of the School of Public Health, Tongji Medical College, and Dongfeng General Hospital. And has been conducted according to the principles expressed in the Declaration of Helsinki. All participants provided written informed consent.

### Data collection

Trained interviewers used a semi-structured questionnaire to collect baseline and follow-up data including demographic information, family and personal disease histories, drug use, lifestyle, and exercise during face-to-face interviews. Height, weight, and waist circumference were measured with participants wearing light indoor clothing and no shoes. BMI was calculated as weight in kilograms divided by height in meters squared. Overweight and obesity were defined according to the classifications for Asian populations as a BMI between 24.0 to 28.0 kg/m^2^ and a BMI ≥ 28.0 kg/m^2^, respectively [17]. Abdominal obesity was defined according to guidelines for Chinese populations as a waist circumference ≥ 85 cm for men and ≥ 80 cm for women [18]. Information including smoking status, drinking status, and exercise was also obtained. The general health examination was performed at the same time. Biochemical parameters including fasting plasma glucose, blood lipids [total cholesterol (TC), triglycerides (TG), high-density lipoprotein cholesterol (HDL-C), and low-density lipoprotein cholesterol (LDL-C)], hepatic function [alanine aminotransferase (ALT) and aspartate aminotransferase (AST)], blood routine, and urine routine were measured at baseline and follow-up. The concentrations of glycosylated hemoglobin and homocysteine, and hepatitis B test were measured at follow-up. The hospital’s laboratory measured fasting plasma glucose with Aeroset automatic analyzer (Abbott, USA). Blood lipids and hepatic function were measured with ARCHITECTCi8200 automatic analyzer (ABBOTT Laboratories. Abbott Park, Illinois, USA) using the Abbott Diagnostic reagents according to the instructions of the manufacturer.

### Assessment of NAFLD

Abdominal ultrasonography at baseline and follow-up was performed with Aplio XG (TOSHIBA, Japan) by experienced technicians who have no knowledge of the study objective. The severity of fatty liver was graded as mild, moderate, or severe according to ultrasound criteria as follows [19]: mild fatty liver is defined as slight diffuse increase in fine echoes in the liver parenchyma with normal visualization of the diaphragm and intrahepatic vessel borders. Moderate fatty liver is defined as moderate diffuse increase in fine echoes with slightly impaired visualization of the intrahepatic vessels and diaphragm. Severe fatty liver is defined as marked increase in fine echoes with poor or no visualization of the intrahepatic vessel borders, diaphragm and posterior portion of the right lobe of the liver. NAFLD was diagnosed if the individuals met the ultrasound criteria for fatty liver, non-drinkers or drinkers with the ethanol intake less than 140 g in men (70 g in women) per week in the past 12 months [20].

We also divided NAFLD participants into three groups according to NAFLD status: Regression group, those with NAFLD at baseline but without NAFLD at follow-up. Development group, those without NAFLD at baseline but with NAFLD at follow-up. Persistence group, those with NAFLD at baseline and follow-up. Non-NAFLD, those without NAFLD at baseline and follow-up.

### Assessment of diabetes

Diabetes was diagnosed according to the 2013 American Diabetes Association criteria [21]: fasting plasma glucose ≥ 7.0 mmol/L, or use of oral hypoglycemic medication and/or insulin, or both. For self-reported, physician diagnosed diabetes, additional information including diagnostic time and treatments were further obtained. Impaired fasting glucose (IFG) was diagnosed with fasting plasma glucose 6.1–6.9 mmol/L according to the World Health Organization criteria [22].

### Statistical analysis

Because the present study aimed to investigate the bidirectional association between NAFLD and T2DM, we described the statistical analysis separately.

#### Analysis 1: Association of NAFLD with the risk of incident T2DM

**Fig 1:**
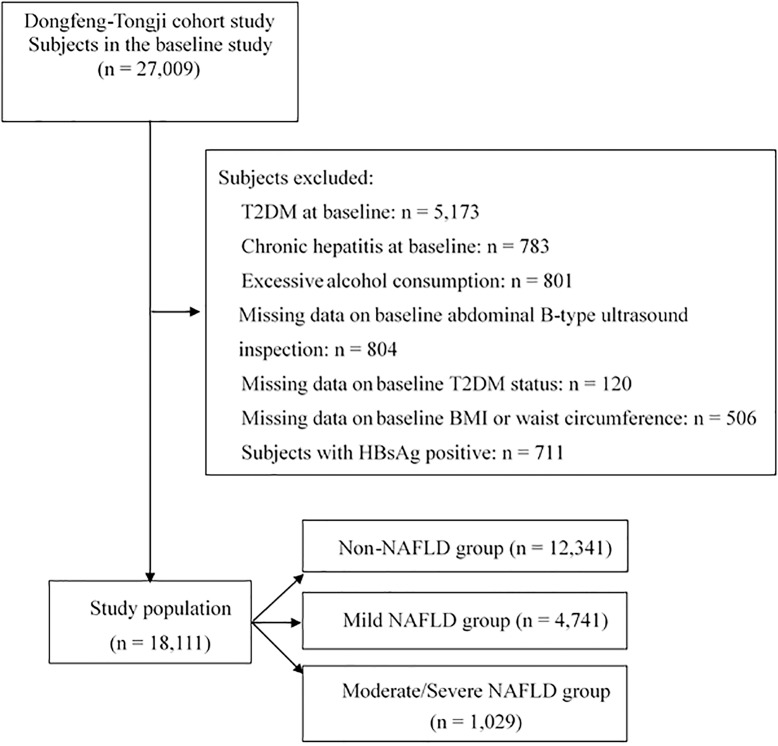
Flow diagram of analysis 1 to investigate the association of NAFLD with incident T2DM risk. NAFLD, nonalcoholic fatty liver disease; T2DM, type 2 diabetes mellitus; BMI, body mass index.

To investigate the association between NAFLD and incident T2DM risk, participants with T2DM (n = 5,173), chronic hepatitis (n = 783), consumption of excessive alcohol (n = 801) at baseline were excluded. Individuals with missing data on abdominal B-type ultrasound inspection (n = 804), T2DM status (n = 120), BMI or waist circumference (n = 506) at baseline were further excluded. Lastly, subjects with HBsAg positive (n = 711) were further excluded. After exclusion, a total of 18,111 participants were included in the final analysis (**Fig 1**). Person-years of follow-up were calculated from the baseline interview to the date diagnosed with diabetes, non-diabetic death or follow-up in 2013, whichever occurred first. Cox proportional hazards regression was used to calculate the hazard ratios (HRs) and 95% confidence intervals (CIs) of T2DM according to the presence and severity of NAFLD at baseline. To assess the interaction of overweight/obesity or abdominal obesity and NAFLD with incident T2DM risk, we introduced the interaction terms into the Cox regression models. In addition, we conducted Cox proportional hazards analysis to examine the combined effects of overweight/obesity, abdominal obesity, and NAFLD on incident T2DM risk. For the multivariate analysis, we adjusted for age (continuous) and sex (male or female) in model 1; in model 2 we further adjusted for lifestyle factors including drinking status (never, ever, or current), smoking status (never, ever, or current) and exercise (yes or no), and family history of diabetes (yes or no); based on model 2, baseline total cholesterol (continuous), triglycerides (continuous), and fasting plasma glucose (continuous) were further adjusted in model 3; in model 4 baseline BMI (continuous) and waist circumference (continuous) were additionally adjusted.

#### Analysis 2: Association of T2DM with the risk of incident NAFLD

**Fig 2:**
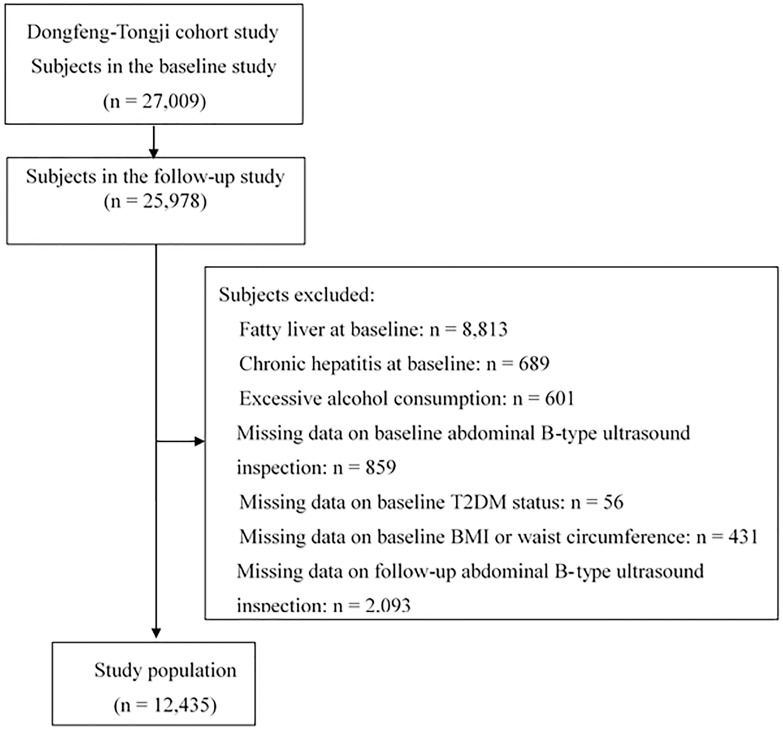
Flow diagram of analysis 2 to investigate the association of T2DM with incident NAFLD risk. T2DM, type 2 diabetes mellitus; NAFLD, nonalcoholic fatty liver disease; BMI, body mass index.

To examine the association between T2DM and incident NAFLD risk, participants with fatty liver (n = 8,813), chronic hepatitis (n = 689), consumption of excessive alcohol (n = 601) at baseline were excluded. Individual with missing data on abdominal B-type ultrasound inspection (n = 859), T2DM status (n = 56), BMI or waist circumference (n = 431) at baseline were further excluded. Those with missing data on abdominal B-type ultrasound inspection (n = 2,093) in the follow-up were also excluded. Finally, 12,435 participants were included in the final analysis (**Fig 2**). The association between IFG or T2DM and incident NAFLD risk was assessed with logistic regression model. Stratified analysis according to overweight/obesity and abdominal obesity was further conducted. Additionally, we also used logistic regression model to investigate the combined effects of overweight/obesity, abdominal obesity, and T2DM on incident NAFLD risk.

For comparisons, we used χ2 tests for categorical variables and analysis of variance for continuous variables. A 2-sided *P* value < 0.05 was used to determine statistical significance. Statistical analyses were performed using SPSS, version 17.0.

## Results

### Analysis 1: Association of NAFLD with the risk of incident T2DM

The baseline clinical and biochemical characteristics of the studied subjects are shown in **Table 1**. With the severity of NAFLD increased, subjects were more likely to be female, less exercise, with higher levels of BMI, waist circumference, fasting plasma glucose, systolic blood pressure, diastolic blood pressure, triglycerides, total cholesterol, LDL cholesterol, AST and ALT levels, in contrast, with lower levels of HDL cholesterol. T2DM incidence in moderate/severe group (18.08%) was significantly higher than those in mild group (10.59%) and normal group (4.65%).

**Table 1:** Baseline characteristics of the subjects according to the NAFLD status.

Variables	Non-NAFLD	NAFLD		*P*-value
		Mild	Moderate/Severe	
N (%)	12341 (68.14)	4741 (26.18)	1029 (5.68)	
Age (years)	63.05 ± 8.07	63.25 ± 7.59	63.11 ± 7.46	0.36
Males, n (%)	5383 (43.62)	1775 (37.44)	370 (35.96)	< 0.001
Family history of diabetes, n (%)	476 (3.96)	176 (3.79)	43 (4.33)	0.71
Smoking, n (%)				< 0.001
Never	8821 (72.01)	3495 (74.28)	777 (76.10)	
Ever	1306 (10.66)	499 (10.61)	119 (11.66)	
Current	2122 (17.32)	711 (15.11)	125 (12.24)	
Drinking, n (%)				0.15
Never	9341 (75.78)	3643 (76.95)	787 (76.63)	
Ever	597 (4.84)	218 (4.60)	61 (5.94)	
Current	2389 (19.38)	873 (18.44)	179 (17.43)	
Exercise, n (%)				< 0.001
Yes	11060 (89.62)	4199 (88.57)	881 (85.62)	
No	1281 (10.38)	542 (11.43)	148 (14.38)	
BMI (kg/m^2^)	23.28 ± 2.89	26.28 ± 2.93	28.28 ± 3.66	< 0.001
Waist circumference (cm)	79.96 ± 8.71	86.88 ± 8.39	92.11 ± 8.99	< 0.001
Fasting plasma glucose (mmol/L)	5.46 ± 0.56	5.67 ± 0.57	5.75 ± 0.60	< 0.001
Systolic blood pressure (mmHg)	127.21 ± 18.44	131.13 ± 18.19	136.07 ± 18.10	< 0.001
Diastolic blood pressure (mmHg)	76.84 ± 10.67	79.12 ± 10.71	83.17 ± 11.12	< 0.001
LDL-C (mmol/L)	2.98 ± 0.78	3.09 ± 0.92	3.11 ± 0.87	< 0.001
HDL-C (mmol/L)	1.48 ± 0.41	1.38 ± 0.37	1.41 ± 0.50	< 0.001
TG (mmol/L)	1.21 ± 0.72	1.71 ± 1.04	1.96 ± 1.25	< 0.001
TC (mmol/L)	5.11 ± 0.93	5.30 ± 0.96	5.37 ± 1.00	< 0.001
ALT (U/L)	21.26 ± 17.75	26.47 ± 19.66	33.73 ± 20.67	< 0.001
AST (U/L)	24.33 ± 11.94	25.33 ± 13.97	29.40 ± 14.92	< 0.001
Incident T2DM, n (%)	574 (4.65)	502 (10.59)	186 (18.08)	< 0.001

BMI, body mass index; LDL-C, low-density lipoprotein cholesterol; HDL-C, high-density lipoprotein cholesterol; TG, triglycerides; TC, total cholesterol; ALT, alanine aminotransferase; AST, aspartate aminotransferase; T2DM, type 2 diabetes mellitus.

Of the 18,111 non-diabetic participants at baseline, 1,262 (6.97%) developed T2DM during the 4.50 ± 0.60 years. The baseline clinical and biochemical characteristics of the study subjects according to the development of T2DM are shown in **S1 Table**. Among those who developed T2DM during the follow-up period, 54.5% of the subjects had NAFLD at baseline, compared with 30.2% in those remaining free of T2DM during the follow-up period (*P* < 0.001).

Association between NAFLD at baseline and incident T2DM risk is shown in **Table 2**. As the severity of NAFLD increased, HR of incident T2DM increased in the fully adjusted model. Participants with mild or moderate/severe NAFLD were at significantly increased incident T2DM risk (HR: 1.88 [95% CI 1.63–2.18] and 2.34 [1.85–2.96], respectively) (*P* for trend < 0.001) compared to participants without NAFLD in the final multivariable model. After further exclusion of current drinkers, the relationship between NAFLD and incident T2DM risk did not materially change (**S2 Table**).

**Table 2:** Association between NAFLD and incident T2DM risk.

	Non-NAFLD	NAFLD		*P*-trend
		Mild	Moderate/Severe	
Cases/person-years	574/55685	502/21242	186/4538	
Incidence density (per 1000 person-years)	10.31	23.63	40.99	
Model 1	1.00	2.39 (2.12–2.69)	4.05 (3.43–4.78)	< 0.001
Model 2	1.00	2.37 (2.10–2.68)	4.02 (3.40–4.76)	< 0.001
Model 3	1.00	2.36 (2.06–2.70)	3.52 (2.87–4.32)	< 0.001
Model 4	1.00	1.88 (1.63–2.18)	2.34 (1.85–2.96)	< 0.001

NAFLD, nonalcoholic fatty liver disease; T2DM, type 2 diabetes mellitus; BMI, body mass index.

Model 1: adjusted for age and sex.

Model 2: adjusted for variables in model 1 plus drinking, smoking, exercise, and family history of diabetes.

Model 3: adjusted for variables in model 2 plus baseline concentrations of fasting plasma glucose, triglycerides, and total cholesterol.

Model 4: adjusted for variables in model 3 plus baseline BMI and waist circumference.

We further examined the association between NAFLD status and the risk of incident T2DM stratified by baseline glycemic status (baseline normal vs. IFG). For those with normal glycemia, participants with mild or moderate/severe NAFLD were at significantly increased incident T2DM risk (HR: 1.81 [95% CI 1.46–2.23] and 2.10 [1.49–2.97], respectively) compared to participants without NAFLD in the final multivariable model. For those with IFG, compared with participants without NAFLD, individuals with mild and moderate/severe NAFLD had a monotonic elevated risk of developing T2DM (HR: 1.29 [95% CI: 1.04–1.59] and 1.55 [1.13–2.14], respectively) after adjustment for potential confounders (**S3 Table**).

We further examined the association between NAFLD and risk of incident IFG and T2DM. Participants with NAFLD were at significantly increased risk of incident IFG (OR: 1.46 [95% CI 1.32–1.62]) and T2DM (HR: 2.54 [95% CI 2.22–2.90]) (**S4 Table**).

The incident T2DM risk based on NAFLD status are shown in **S5 Table**. Participants in the Regression NAFLD group and Development/Persistence NAFLD group were at significantly increased incident T2DM risk (HR: 1.46 [95% CI 1.15–1.84] and 2.84 [2.41–3.36], respectively) (*P* for trend < 0.001) compared with non-NAFLD participants in the final multivariate model.

**Fig 3:**
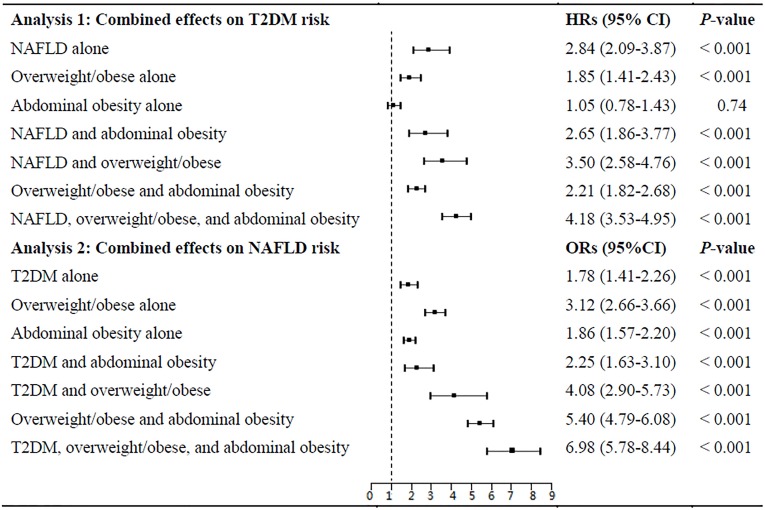
Combined effects of NAFLD, overweight/obesity, and abdominal obesity on the incident T2DM risk and combined effects of T2DM, overweight/obesity, and abdominal obesity on incident NAFLD risk. Cox proportional hazards regression was used to calculate the hazard ratio (HR) and its 95% CI of incident diabetes. Logistic regression analysis was used to calculate the odds ratio (OR) and its 95% CI of incident NAFLD. Age, sex, drinking, smoking, exercise, and family history of diabetes were adjusted in the models.

We further examined the combined effects of NAFLD, overweight/obesity, and abdominal obesity on incident T2DM risk (**Fig 3**). Participants with the NAFLD, overweight/obesity, and abdominal obesity concurrently had 4.18 folds risk of T2DM (95% CI: 3.53–4.95). No significant interactions were found for the NAFLD-obesity on incident T2DM risk (all *P*-interaction > 0.05; **S1 Fig**).

### Analysis 2: Association of T2DM with the risk of incident NAFLD

The characteristics of the subjects according to the T2DM status are shown in **Table 3.** Compared with those with normal fasting plasma glucose, individuals with IFG or T2DM were more likely to be older, with higher levels of BMI, waist circumference, fasting plasma glucose, systolic blood pressure, triglycerides, total cholesterol, LDL cholesterol and lower levels of HDL cholesterol at baseline. The incident NAFLD in normal group, IFG group, and T2DM group were 20.11%, 24.98% and 27.85%, respectively.

**Table 3:** Baseline characteristics of the subjects according to the T2DM status.

Variables	Normal	IFG	T2DM	*P*-value
N (%)	9385 (75.47)	1373 (11.04)	1677 (13.49)	
Age (years)	62.18 ± 7.81	64.42 ± 7.37	65.26 ± 7.36	< 0.001
Male, n (%)	3846 (40.98)	756 (55.06)	773 (46.09)	< 0.001
Family history of diabetes, n (%)	368 (4.03)	53 (3.94)	205 (12.37)	< 0.001
Smoking, n (%)				< 0.001
Never	6831 (73.33)	941 (68.84)	1248 (75.05)	
Ever	895 (9.61)	203 (14.85)	193 (11.61)	
Current	1590 (17.07)	223 (16.31)	222 (13.35)	
Drinking, n (%)				< 0.001
Never	7138 (76.16)	979 (71.30)	1307 (77.98)	
Ever	425 (4.53)	83 (6.05)	126 (7.52)	
Current	1809 (19.30)	311 (22.65)	243 (14.50)	
Exercise, n (%)				0.17
Yes	8426 (89.78)	1255 (91.41)	1511 (90.10)	
No	959 (10.22)	118 (8.59)	166 (9.90)	
BMI (kg/m^2^)	23.25 ± 2.84	23.76 ± 2.84	23.91 ± 2.74	< 0.001
Waist circumference (cm)	79.69 ± 8.58	81.03 ± 8.33	82.44 ± 8.03	< 0.001
Fasting plasma glucose (mmol/L)	5.31 ± 0.44	6.38 ± 0.24	7.82 ± 2.62	< 0.001
Systolic blood pressure (mmHg)	126.55 ± 18.33	129.92 ± 17.43	132.31 ± 18.20	< 0.001
Diastolic blood pressure (mmHg)	76.94 ± 10.55	76.74 ± 10.54	76.02 ± 10.50	0.004
LDL-C (mmol/L)	2.97 ± 0.77	3.04 ± 0.76	3.02 ± 0.83	0.003
HDL-C (mmol/L)	1.48 ± 0.40	1.46 ± 0.40	1.39 ± 0.36	< 0.001
TG (mmol/L)	1.18 ± 0.68	1.31 ± 0.96	1.37 ± 0.82	< 0.001
TC (mmol/L)	5.08 ± 0.91	5.19 ± 0.95	5.12 ± 0.99	< 0.001
ALT (U/L)	21.37 ± 18.75	22.66 ± 16.11	22.91 ± 18.53	0.001
AST (U/L)	24.49 ± 12.56	24.63 ± 11.05	23.10 ± 10.01	< 0.001
Incident NAFLD, n (%)	1887 (20.11)	343 (24.98)	467 (27.85)	< 0.001

IFG, impaired fasting glucose; BMI, body mass index; T2DM, type 2 diabetes mellitus; LDL-C, low-density lipoprotein cholesterol; HDL-C, high-density lipoprotein cholesterol; TG, triglycerides; TC, total cholesterol; ALT, alanine aminotransferase; AST, aspartate aminotransferase; NAFLD, nonalcoholic fatty liver disease.

In the parallel analysis to investigate the association between T2DM with incident NAFLD risk, 2,697 (21.7%) participants were newly diagnosed with NAFLD during the follow-up period. The characteristics of the study populations according to the NAFLD group are shown in **S6 Table**.

The association between IFG or T2DM and incident NAFLD risk is shown in **Table 4**. Compared with those without IFG or T2DM, the ORs (95% CIs) of NAFLD in subjects with IFG or T2DM were 1.35 (1.16–1.57) and 1.40 (1.22–1.62), respectively (*P* for trend < 0.001) in the final multivariable model. The association between T2DM and incident NAFLD risk remained significant after exclusion of the current drinkers (**S7 Table**).

**Table 4:** Associations between IFG, diabetes and incident NAFLD risk.

	Normal	IFG	T2DM	*P*-trend
NAFLD, n (%)	1887 (20.11)	343 (24.98)	467 (27.85)	
Model 1	1.00	1.44 (1.26–1.65)	1.62 (1.43–1.82)	< 0.001
Model 2	1.00	1.46 (1.27–1.67)	1.64 (1.45–1.86)	< 0.001
Model 3	1.00	1.42 (1.23–1.64)	1.52 (1.33–1.74)	< 0.001
Model 4	1.00	1.35 (1.16–1.57)	1.40 (1.22–1.62)	< 0.001

NAFLD, nonalcoholic fatty liver disease; IFG, impaired fasting glucose; T2DM, type 2 diabetes mellitus; BMI, body mass index.

Model 1: adjusted for age and sex.

Model 2: adjusted for variables in model 1 plus drinking, smoking, exercise, and family history of diabetes.

Model 3: adjusted for variables in model 2 plus baseline concentrations of triglycerides and total cholesterol.

Model 4: adjusted for variables in model 3 plus baseline BMI and waist circumference.

We further examined the combined effects of T2DM, overweight/obesity, and abdominal obesity on the incident NAFLD risk (**Fig 3**). The combination of T2DM, overweight/obesity, and abdominal obesity markedly increased the risk of incident NAFLD (OR: 6.98 [95% CI: 5.78–8.44]) after adjustment for age, sex, smoking, drinking, exercise, family history of diabetes. However, we did not observe the diabetes-obesity interactions on the incident NAFLD risk (**S1 Fig**).

## Discussion

The present study examined the bidirectional longitudinal association between NAFLD and T2DM in a prospective cohort among a middle-aged and elderly Chinese population. The results provided growing evidence that NAFLD and T2DM were closely related to each other, independently of potential confounders. In addition, there were combined effects of NAFLD, overweight/obesity, and abdominal obesity on incident T2DM risk, vice versa.

### Analysis 1: Association of NAFLD with the risk of incident T2DM

The present study found that NAFLD was independently associated with increased incident T2DM risk. In addition, the incident T2DM risk increased with the severity of NAFLD. The overall estimate of the risk of incident T2DM in those with NAFLD was similar as previous studies [23,24].

To the best of our knowledge, several longitudinal studies investigated the association between NAFLD diagnosed by ultrasonography and incident T2DM risk [23,24]. A systematic review and meta-analysis of twenty prospective studies reported that NAFLD was associated with an increased risk of incident T2DM with a pooled relative risk of 1.97 for alanine aminotransferase, 1.58 for aspartate aminotransferase, and 1.86 for ultrasonography, respectively [7]. The present study provided further evidence to the association between NAFLD and incident T2DM risk among middle-aged and elderly Chinese population.

Because overweight/obesity, abdominal obesity, and NAFLD often coexist in cluster, we further investigated their combined effects on incident T2DM risk. Participants with NAFLD, overweight/obesity, and abdominal obesity concurrently had the strongest effects on incident T2DM risk. Among the three risk factors of incident diabetes, NAFLD alone had the strongest association with incident T2DM risk (fully adjusted HR for NAFLD alone: 2.84[2.09–3.87]). Overweight/obesity was an independent risk factor for incident T2DM. However, participant with abdominal obesity alone was not associated with incident T2DM risk in the present middle-aged and elderly population. This might be due to that these participants with relatively smaller waist circumference (85.8 cm), compared with those with overweight/obesity, abdominal obesity, and NAFLD concurrently (91.4 cm). In addition, previous study indicated that indiviudals with or without abdominal obesity had similar incidence of T2DM when they had multiple metabolic syndrome components concurrently [25]. Overweight/obesity may increase fat accumulation in liver and subsequently cause fatty liver. Fat accumulation in hepatic may decrease insulin activation of glycogen synthase and increase gluconeogenesis, and subsequently lead to T2DM [26].

Several potential mechanisms might be involved in the association of NAFLD and incident T2DM risk. First, NAFLD is characterized by a large number of hepatic triglyceride which could result in dysfunctional lipid metabolism and disordered glucose regulation[6]. Second, NAFLD could promote insulin resistance of hepatic, skeletal muscle, and adipose tissues [27]. Additionally, insulin resistance increases the transportation of free tatty acid to liver and increases hepatic fatty acid β oxidation [28]. Third, compared to those without NAFLD, subjects with NAFLD have higher levels of inflammatory markers which are known risk factors for diabetes [29].

### Analysis 2: Association of T2DM with the risk of incident NAFLD

In the present study, we found that T2DM was associated with increased incident NAFLD risk after adjustment for potential covariates. Compared with participants with normal glycemia, individuals with T2DM have 1.40 folds risk of incident NAFLD. In addition, T2DM, overweight/ obesity, and abdominal obesity had combined effects on incident NAFLD risk.

Several cross-sectional studies showed that T2DM patients have an increased prevalence of NAFLD and diabetic status were considered to be a significant risk factor for advanced fibrosis in patients with NAFLD [30]. Other longitudinal studies also demonstrated that diabetes was an independent risk factor for hepatocellular carcinoma and serious liver disease [12,31]. In the present prospective study, we found that subjects with IFG or T2DM had consistently increased risk of incident NAFLD, independent of other potential confounders including baseline BMI, waist circumference, triglycerides and total cholesterol levels. Further exclusion of the current drinkers did not significantly alter the positive association between T2DM and incident NAFLD risk.

Furthermore, T2DM, overweight/obesity, and abdominal obesity had combined effects on incident NAFLD risk. To our knowledge, this is the first prospective study to assess the combined effects of these three factors on the incident NAFLD risk. Among the three risk factors of incident NAFLD, overweight/obesity alone had the strongest and T2DM had the weakest association with incident NAFLD risk. Previous study indicated that subjects with diabetes alone or obesity alone was more susceptible to develop serious liver disease [12]. The Framingham Offspring Study findings indicated that the combination of diabetes and abdominal obesity posed a higher risk for stroke than either condition alone [32], similar as the present study. Further prospective studies are needed to validate these findings and to investigate the potential mechanisms of these associations.

Growing evidence suggested that there was bidirectional association between NAFLD with MetS and T2DM [14–16]. A series of prospective studies showed that NAFLD strongly increased MetS and T2DM incident risk [7,33]. In addition, several longitudinal studies explored the association between NAFLD development/improvement/persistence and risk of incident T2DM [9,34,35]. Meanwhile, other longitudinal studies indicated that MetS and T2DM were risk factors for the development of NAFLD [36,37]. NAFLD may be both a cause and a consequence of the MetS and T2DM.

The bidirectional association of NAFLD and T2DM is likely due to “common soil” they shared. Firstly, insulin resistance is an established risk factor for both conditions and plays a role in the interaction between NAFLD and T2DM [6]. Studies suggested that insulin resistance is associated with advanced fibrosis of NAFLD [38]. However, insulin sensitizers do not reverse fibrosis although it is associated with improved grade of steatosis, suggesting that insulin resistance might be necessary but not sufficient in the development of NAFLD [39]. Secondly, disordered lipid metabolism, increased oxidative stress and inflammation contribute to both entities[40]. Thirdly, some genetic and environmental factors might also contribute to both NAFLD and T2DM [41].

This study investigated the complex bidirectional association between NAFLD and T2DM with relative large sample size in a population-based prospective cohort. Moreover, to investigate the association of NAFLD with incident T2DM risk, time-dependent Cox regression models were used, which minimized the possibility of residual and time-dependent confounding. Consequently, we could simultaneously examine the two temporal hypotheses of whether NAFLD is an independent risk factor for T2DM and, vice versa, whether T2DM increases incident NAFLD risk.

Several limitations should be considered. Firstly, the present cohort study was conducted among a middle-aged and elderly Chinese population, thus the results might not be generalized to other populations. Secondly, lack of a 2-h oral glucose tolerance test limited us to exclude undiagnosed diabetes at baseline and identify new-onset cases during the follow-up. Thirdly, liver biopsy is gold standard to diagnose NAFLD [42]. However, in the present study we did not perform liver biopsy but ultrasonography, which is a sensitive and feasible surrogate measure in large populations. A meta-analysis also indicated that ultrasonography has a sensitivity of 84.8% and a specificity 93.6% for screening purposes [43] and another study indicated that semi-quantitative ultrasonography indices had more advantages [44]. Fourthly, we used hepatitis B virus serological markers in 2013 to distinguish participants with or without HBsAg infection because these data were not available at baseline, this might slightly bias our results. Finally, we did not collect information about hepatitis C infection, however, in the present study we did exclude subjects with history of chronic hepatitis.

## Conclusions

In conclusion, the present prospective cohort study provides evidence that the association between NAFLD and T2DM is bidirectional. Future studies are needed to investigate the potential mechanisms. Given that both NAFLD and T2DM are common in the worldwide, our findings may provide important public health implications for the prevention and management of both conditions.

## Supporting information

S1 Fig Stratified analysis by the overweight/obesity and abdominal obesity. (DOCX)

S1 Table Baseline characteristics of the subjects according to the incident diabetes status. (DOCX)

S2 Table Association between NAFLD and incident T2DM risk among non-drinkers. (DOCX)

S3 Table Association between NAFLD and incident T2DM risk according to baseline glycemic status. (DOCX)

S4 Table Association between NAFLD and incident IFG and T2DM risk. (DOCX)

S5 Table Association between NAFLD status and incident T2DM risk. (DOCX)

S6 Table Baseline characteristics of the subjects according to the incident NAFLD status. (DOCX)

S7 Table Associations between IFG, T2DM and incident NAFLD risk among non-drinkers. (DOCX)
