# Supplementary material for: Bidirectional association between nonalcoholic fatty liver disease and type 2 diabetes in Chinese population: Evidence from the Dongfeng-Tongji cohort study
Source: PLoS One. 2017 Mar 28;12(3):e0174291. doi: 10.1371/journal.pone.0174291 (PMC5369778; doi:10.1371/journal.pone.0174291)
Supplement: S1 Fig — (DOCX) [file pone.0174291.s001.docx]

**S1 Fig Stratified analysis by the overweight/obesity and abdominal obesity**

| **Subgroups** |  | **HRs (95% CIs)** | ***P*-value** | ***P-*interaction** |
| --- | --- | --- | --- | --- |
| \| **NAFLD with incident T2DM risk** \| \| --- \| |  |  |  |  |
| Overweight/obesity ^a^ |  |  |  | 0.41 |
| No |  | 1.58 (1.18-2.11) | 0.002 |  |
| Yes |  | 1.53 (1.31-1.79) | < 0.001 |  |
| Abdominal obesity ^b^ |  |  |  | 0.46 |
| No |  | 1.72 (1.32-2.24) | < 0.001 |  |
| Yes |  | 1.64 (1.40-1.93) | < 0.001 |  |
| **T2DM with incident NAFLD risk** |  | **ORs (95% CIs)** | ***P-*value** | ***P-*interaction** |
| Overweight/obesity ^c^ |  |  |  | 0.25 |
| No |  | 1.45 (1.17-1.80) | < 0.001 |  |
| Yes |  | 1.32 (1.11-1.58) | 0.002 |  |
| Abdominal obesity ^d^ | 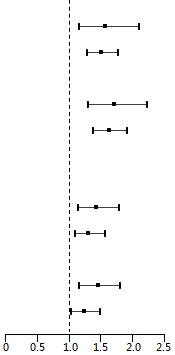 |  |  | 0.12 |
| No |  | 1.47 (1.18-1.82) | < 0.001 |  |
| Yes |  | 1.26 (1.06-1.50) | 0.009 |  |
|  |  |  |  |  |

NAFLD, nonalcoholic fatty liver disease; T2DM, type 2 diabetes mellitus; HRs, hazard ratios; ORs, odd ratios; CIs, confidence intervals.

Cox proportional hazards regression was used in the analysis of NAFLD with incident T2DM risk, HRs were estimated and their 95% CI were determined. Logistic regression model was used in the analysis of T2DM with incident NAFLD risk, ORs were estimated and their 95% CIs were determined.

^a^ Adjusted for age (continuous), sex (male/female), drinking (never, ever, or current), smoking (never, ever, or current), exercise (yes/no), and family history of diabetes (yes/no), fasting plasma glucose (continuous), triglycerides (continuous) , total cholesterol (continuous), and waist circumference (continuous).

^b^ Adjusted for age (continuous), sex (male/female), drinking (never, ever, or current), smoking (never, ever, or current), exercise (yes/no), and family history of diabetes (yes/no), fasting plasma glucose (continuous), triglycerides (continuous) , total cholesterol (continuous), and body mass index (continuous).

^c^ Adjusted for age (continuous), sex (male/female), drinking (never, ever, or current), smoking (never, ever, or current), exercise (yes/no), and family history of diabetes (yes/no), triglycerides (continuous) , total cholesterol (continuous), and waist circumference (continuous).

^d^ Adjusted for age (continuous), sex (male/female), drinking (never, ever, or current), smoking (never, ever, or current), exercise (yes/no), and family history of diabetes (yes/no), triglycerides (continuous) , total cholesterol (continuous), and body mass index (continuous).
