# Supplementary material for: Bidirectional association between nonalcoholic fatty liver disease and type 2 diabetes in Chinese population: Evidence from the Dongfeng-Tongji cohort study
Source: PLoS One. 2017 Mar 28;12(3):e0174291. doi: 10.1371/journal.pone.0174291 (PMC5369778; doi:10.1371/journal.pone.0174291)
Supplement: S1 Table — (DOCX) [file pone.0174291.s002.docx]

**S1 Table Baseline characteristics of the subjects according to the incident diabetes status**

| **Variables** | **Non-T2DM** | **T2DM** | ***P*-value** |
| --- | --- | --- | --- |
| N (%) | 16849 (93.03) | 1262 (6.97) |  |
| Age (years) | 63.11 ± 7.96 | 63.07 ± 7.36 | 0.85 |
| Male, n (%) | 6981 (41.43) | 547 (43.34) | 0.18 |
| Family history of diabetes, n (%) | 632 (3.85) | 63 (5.09) | 0.03 |
| Smoking, n (%) |  |  | 0.47 |
| Never | 12199 (72.95) | 894 (71.41) |  |
| Ever | 1780 (10.64) | 144 (11.50) |  |
| Current | 2744 (16.41) | 214 (17.09) |  |
| Drinking, n (%) |  |  | 0.003 |
| Never | 12855 (76.40) | 916 (72.58) |  |
| Ever | 796 (4.73) | 80 (6.34) |  |
| Current | 3175 (18.87) | 266 (21.08) |  |
| Exercise, n (%) |  |  | 0.37 |
| Yes | 15025 (89.17) | 1115 (88.35) |  |
| No | 1824 (10.83) | 147 (11.65) |  |
| BMI (kg/m^2^) | 24.23 ± 3.34 | 25.89 ± 3.32 | < 0.001 |
| Waist circumference (cm) | 82.16 ± 9.38 | 86.44 ± 9.52 | < 0.001 |
| Fasting plasma glucose (mmol/L) | 5.50 ± 0.56 | 6.01 ± 0.61 | < 0.001 |
| Systolic blood pressure (mmHg) | 128.44 ± 18.48 | 132.74 ± 18.55 | < 0.001 |
| Diastolic blood pressure (mmHg) | 77.66 ± 10.81 | 79.68 ± 10.93 | < 0.001 |
| LDL-C (mmol/L) | 3.02 ± 0.83 | 3.03 ± 0.77 | 0.58 |
| HDL-C (mmol/L) | 1.45 ± 0.40 | 1.40 ± 0.45 | < 0.001 |
| TG (mmol/L) | 1.36 ± 0.86 | 1.66 ± 1.18 | < 0.001 |
| TC (mmol/L) | 5.17 ± 0.95 | 5.25 ± 0.93 | 0.005 |
| ALT (U/L) | 22.94 ± 18.31 | 28.56 ± 23.40 | < 0.001 |
| AST (U/L) | 24.74 ± 12.41 | 26.62 ± 16.57 | < 0.001 |
| NAFLD, n (%) | 5082 (30.16) | 688 (54.52) | < 0.001 |

T2DM, type 2 diabetes mellitus; BMI, body mass index; LDL-C, low-density lipoprotein cholesterol; HDL-C, high-density lipoprotein cholesterol; TG, triglycerides; TC, total cholesterol; ALT, alanine aminotransferase; AST, aspartate aminotransferase; NAFLD, nonalcoholic fatty liver disease.
