# Supplementary material for: Bidirectional association between nonalcoholic fatty liver disease and type 2 diabetes in Chinese population: Evidence from the Dongfeng-Tongji cohort study
Source: PLoS One. 2017 Mar 28;12(3):e0174291. doi: 10.1371/journal.pone.0174291 (PMC5369778; doi:10.1371/journal.pone.0174291)
Supplement: S2 Table — (DOCX) [file pone.0174291.s003.docx]

**S2 Table Association between NAFLD and incident T2DM risk among non-drinkers**

|  | Non-NAFLD | NAFLD | | *P*-trend |
| --- | --- | --- | --- | --- |
|  |  | Mild | Moderate/Severe |  |
| Cases/person-years | 453/44936 | 392/17362 | 151/3758 |  |
| Incidence density (per 1000 person-years) | 10.08 | 22.58 | 40.18 |  |
| Model 1 | 1.00 | 2.39 (2.12-2.69) | 4.05 (3.43-4.78) | <0.001 |
| Model 2 | 1.00 | 2.37 (2.10-2.68) | 4.02 (3.40-4.76) | <0.001 |
| Model 3 | 1.00 | 2.36 (2.06-2.70) | 3.52 (2.87-4.32) | <0.001 |
| Model 4 | 1.00 | 1.88 (1.63-2.18) | 2.34 (1.85-2.96) | <0.001 |

NAFLD, nonalcoholic fatty liver disease; T2DM, type 2 diabetes mellitus; BMI, body mass index.

Model 1: adjusted for age and sex.

Model 2: adjusted for variables in model 1 plus smoking, exercise, and family history of diabetes.

Model 3: adjusted for variables in model 2 plus baseline concentrations of fasting plasma glucose, triglycerides, and total cholesterol.

Model 4: adjusted for variables in model 3 plus baseline BMI and waist circumference.
