# Supplementary material for: Bidirectional association between nonalcoholic fatty liver disease and type 2 diabetes in Chinese population: Evidence from the Dongfeng-Tongji cohort study
Source: PLoS One. 2017 Mar 28;12(3):e0174291. doi: 10.1371/journal.pone.0174291 (PMC5369778; doi:10.1371/journal.pone.0174291)
Supplement: S3 Table — (DOCX) [file pone.0174291.s004.docx]

**S3 Table Association between NAFLD and incident T2DM risk according to baseline glycemic status**

| Glycemic status | Non-NAFLD | NAFLD | | *P*-trend |
| --- | --- | --- | --- | --- |
|  |  | Mild | Moderate/Severe |  |
| **Normal** |  |  |  |  |
| Model 1 | 1.00 | 2.35 (2.00-2.76) | 4.28 (3.43-5.34) | < 0.001 |
| Model 2 | 1.00 | 2.35 (2.00-2.76) | 4.30 (3.43-5.39) | < 0.001 |
| Model 3 | 1.00 | 2.34 (1.93-2.83) | 3.30 (2.42-4.50) | < 0.001 |
| Model 4 | 1.00 | 1.81 (1.46-2.23) | 2.10 (1.49-2.97) | 0.006 |
| **IFG** |  |  |  |  |
| Model 1 | 1.00 | 1.47 (1.22-1.76) | 2.06 (1.60-2.66) | < 0.001 |
| Model 2 | 1.00 | 1.44 (1.20-1.74) | 2.03 (1.56-2.62) | < 0.001 |
| Model 3 | 1.00 | 1.46 (1.20-1.77) | 1.97 (1.49-2.60) | < 0.001 |
| Model 4 | 1.00 | 1.29 (1.04-1.59) | 1.55 (1.13-2.14) | 0.004 |

NAFLD, nonalcoholic fatty liver disease; IFG, impaired fasting glucose; T2DM, type 2 diabetes mellitus; BMI, body mass index.

Model 1: adjusted for age and sex.

Model 2: adjusted for variables in model 1 plus drinking, smoking, exercise, and family history of diabetes.

Model 3: adjusted for variables in model 2 plus baseline concentrations of, triglycerides, and total cholesterol.

Model 4: adjusted for variables in model 3 plus baseline BMI and waist circumference.
