# Supplementary material for: Bidirectional association between nonalcoholic fatty liver disease and type 2 diabetes in Chinese population: Evidence from the Dongfeng-Tongji cohort study
Source: PLoS One. 2017 Mar 28;12(3):e0174291. doi: 10.1371/journal.pone.0174291 (PMC5369778; doi:10.1371/journal.pone.0174291)
Supplement: S4 Table — (DOCX) [file pone.0174291.s005.docx]

**S4** **Table Association between NAFLD and incident IFG and T2DM risk**

|  | Non-NAFLD | NAFLD | *P*-value |
| --- | --- | --- | --- |
| **IFG** |  |  |  |
| Model 1 | 1.00 | 1.51 (1.40-1.63) | < 0.001 |
| Model 2 | 1.00 | 1.51 (1.39-1.63) | < 0.001 |
| Model 3 | 1.00 | 1.53 (1.40-1.68) | < 0.001 |
| Model 4 | 1.00 | 1.46 (1.32-1.62) | < 0.001 |
| **T2DM** |  |  |  |
| Model 1 | 1.00 | 2.89 (2.59-3.23) | < 0.001 |
| Model 2 | 1.00 | 2.56 (2.27-2.89) | < 0.001 |
| Model 3 | 1.00 | 2.54 (2.25-2.87) | < 0.001 |
| Model 4 | 1.00 | 2.54 (2.22-2.90) | < 0.001 |

NAFLD, nonalcoholic fatty liver disease. IFG, impaired fasting glucose; T2DM, type 2 diabetes mellitus; BMI, body mass index.

Model 1: adjusted for age and sex.

Model 2: adjusted for variables in model 1 plus drinking, smoking, exercise, and family history of diabetes.

Model 3: adjusted for variables in model 2 plus baseline concentrations of fasting plasma glucose, triglycerides, and total cholesterol.

Model 4: adjusted for variables in model 3 plus baseline BMI and waist circumference.
