# Supplementary material for: Bidirectional association between nonalcoholic fatty liver disease and type 2 diabetes in Chinese population: Evidence from the Dongfeng-Tongji cohort study
Source: PLoS One. 2017 Mar 28;12(3):e0174291. doi: 10.1371/journal.pone.0174291 (PMC5369778; doi:10.1371/journal.pone.0174291)
Supplement: S5 Table — (DOCX) [file pone.0174291.s006.docx]

**S5 Table Association between NAFLD status and incident T2DM risk**

|  | Non-NAFLD | NAFLD | | *P*-trend |
| --- | --- | --- | --- | --- |
|  |  | Regression | Development/Persistence |  |
| Cases/person-years | 331/37358 | 134/7672 | 746/23969 |  |
| Incidence density (per 1000 person-years) | 8.86 | 17.47 | 31.12 |  |
| Model 1 | 1.00 | 1.92 (1.57-2.34) | 3.85 (3.38-4.39) | < 0.001 |
| Model 2 | 1.00 | 1.80 (1.47-2.22) | 3.86 (3.38-4.40) | < 0.001 |
| Model 3 | 1.00 | 1.77 (1.41-2.21) | 3.58 (3.09-4.16) | < 0.001 |
| Model 4 | 1.00 | 1.46 (1.15-1.84) | 2.84 (2.41-3.36) | < 0.001 |

NAFLD, nonalcoholic fatty liver disease; T2DM, type 2 diabetes mellitus; BMI, body mass index.

Model 1: adjusted for age and sex.

Model 2: adjusted for variables in model 1 plus smoking, exercise, and family history of diabetes.

Model 3: adjusted for variables in model 2 plus baseline concentrations of fasting plasma glucose, triglycerides, and total cholesterol.

Model 4: adjusted for variables in model 3 plus baseline BMI and waist circumference.
