# Supplementary material for: Bidirectional association between nonalcoholic fatty liver disease and type 2 diabetes in Chinese population: Evidence from the Dongfeng-Tongji cohort study
Source: PLoS One. 2017 Mar 28;12(3):e0174291. doi: 10.1371/journal.pone.0174291 (PMC5369778; doi:10.1371/journal.pone.0174291)
Supplement: S6 Table — (DOCX) [file pone.0174291.s007.docx]

**S6 Table Baseline characteristics of the subjects according to the incident NAFLD status**

| **Variables** | **Non-NAFLD** | **NAFLD** | ***P*-value** |
| --- | --- | --- | --- |
| N (%) | 9738 (78.31) | 2697 (21.69) |  |
| Age (years) | 63.04 ± 7.82 | 62.14 ± 7.65 | < 0.001 |
| Males, n (%) | 4449 (45.69) | 926 (34.33) | < 0.001 |
| Family history of diabetes, n (%) | 465 (4.89) | 161 (6.13) | 0.01 |
| Smoking, n (%) |  |  | < 0.001 |
| Never | 6919 (71.59) | 2101 (78.37) |  |
| Ever | 1066 (11.03) | 225 (8.39) |  |
| Current | 1680 (17.38) | 355 (13.24) |  |
| Drinking, n (%) |  |  | 0.002 |
| Never | 7314 (75.19) | 2110 (78.35) |  |
| Ever | 518 (5.32) | 116 (4.31) |  |
| Current | 1896 (19.49) | 467 (17.34) |  |
| Exercise, n (%) |  |  | 0.001 |
| Yes | 8810 (90.47) | 2382 (88.32) |  |
| No | 928 (9.53) | 315 (11.68) |  |
| BMI (kg/m^2^) | 22.93 ± 2.70 | 25.08 ± 2.69 | < 0.001 |
| Waist circumference (cm) | 79.17 ± 8.37 | 83.96 ± 8.07 | < 0.001 |
| Fasting plasma glucose (mmol/L) | 5.75 ± 1.38 | 5.90 ± 1.29 | < 0.001 |
| Systolic blood pressure (mmHg) | 127.16 ± 18.34 | 129.64 ± 18.17 | < 0.001 |
| Diastolic blood pressure (mmHg) | 76.46 ± 10.52 | 78.03 ± 10.56 | < 0.001 |
| LDL-C (mmol/L) | 2.97 ± 0.77 | 3.05 ± 0.81 | < 0.001 |
| HDL-C (mmol/L) | 1.48 ± 0.40 | 1.42 ± 0.38 | < 0.001 |
| TG (mmol/L) | 1.17 ± 0.71 | 1.43 ± 0.81 | < 0.001 |
| TC (mmol/L) | 5.08 ± 0.93 | 5.19 ± 0.93 | < 0.001 |
| ALT (U/L) | 21.44 ± 19.39 | 22.84 ± 14.30 | < 0.001 |
| AST (U/L) | 24.48 ± 12.83 | 23.73 ± 8.71 | 0.007 |
| IFG, n (%) | 1030 (10.58) | 343 (12.72) | < 0.001 |
| T2DM, n (%) | 1210 (12.43) | 467 (17.32) | < 0.001 |

NAFLD, nonalcoholic fatty liver disease; BMI, body mass index; LDL-C, low-density lipoprotein cholesterol; HDL-C, high-density lipoprotein cholesterol; TG, triglycerides; TC, total cholesterol; ALT, alanine aminotransferase; AST, aspartate aminotransferase; IFG, impaired fasting glucose; T2DM, type 2 diabetes mellitus.
