# Supplementary material for: Bidirectional association between nonalcoholic fatty liver disease and type 2 diabetes in Chinese population: Evidence from the Dongfeng-Tongji cohort study
Source: PLoS One. 2017 Mar 28;12(3):e0174291. doi: 10.1371/journal.pone.0174291 (PMC5369778; doi:10.1371/journal.pone.0174291)
Supplement: S7 Table — (DOCX) [file pone.0174291.s008.docx]

**S7 Table Associations between IFG, T2DM and incident NAFLD risk among non-drinkers**

|  | **Normal** | **IFG** | **T2DM** | ***P*-trend** |
| --- | --- | --- | --- | --- |
| NAFLD, n (%) | 1569 (20.71) | 263 (24.76) | 398 (27.75) |  |
| Model 1 | 1.00 | 1.37 (1.18-1.60) | 1.57 (1.38-1.79) | <0.001 |
| Model 2 | 1.00 | 1.39 (1.19-1.62) | 1.58 (1.39-1.81) | <0.001 |
| Model 3 | 1.00 | 1.35 (1.15-1.59) | 1.48 (1.28-1.72) | <0.001 |
| Model 4 | 1.00 | 1.26 (1.06-1.50) | 1.38 (1.18-1.61) | <0.001 |

NAFLD, nonalcoholic fatty liver disease; IFG, impaired fasting glucose; T2DM, type 2 diabetes mellitus; BMI, body mass index.

Model 1: adjusted for age and sex.

Model 2: adjusted for variables in model 1 plus smoking, exercise, and family history of diabetes.

Model 3: adjusted for variables in model 2 plus baseline triglycerides and total cholesterol.

Model 4: adjusted for variables in model 3 plus baseline BMI and waist circumference.
